# Successful radio-frequency catheter ablation of two cases of supraventricular tachycardia via a left-sided inferior vena cava

**DOI:** 10.12669/pjms.36.6.2947

**Published:** 2020

**Authors:** Romana Asad Awan, Muhammad Faisal Khanzada, Zubair Mumtaz, Faisal Qadir

**Affiliations:** 1 Dr. Romana Asad Awan, FCPS. Department of Cardiac Electrophysiology, National Institute of Cardiovascular Diseases, Karachi, Pakistan; 2 Dr. Muhammad Faisal Khanzada, FCPS. Department of Cardiac Electrophysiology, National Institute of Cardiovascular Diseases, Karachi, Pakistan; 3 Dr. Zubair Mumtaz, FCPS. Department of Cardiac Electrophysiology, National Institute of Cardiovascular Diseases, Karachi, Pakistan; 4 Dr. Faisal Qadir, FCPS, Department of Cardiac Electrophysiology, National Institute of Cardiovascular Diseases, Karachi, Pakistan

**Keywords:** Electrophysiology study, Catheter ablation, Inferior vena cava anomaly

## Abstract

Congenital venous anomalies are uncommon, incidental findings encountered during adult interventional electrophysiology procedures. Femoral venous access is conventionally used during cardiac electrophysiology studies to gain access to the heart. The chance finding of an inferior vena cava anomaly may preclude the performance of these procedures from the femoral approach. We describe two cases in which we were able to successfully perform different radiofrequency catheter ablation procedures in the presence of an unusual venous anomaly, the left-sided IVC.

## INTRODUCTION

Diagnostic cardiac electrophysiological studies and radiofrequency catheter ablation are standard curative procedures for a variety of supraventricular and ventricular arrhythmias. Femoral vein access is the preferred approach for advancing multiple catheters via the inferior vena cava (IVC) to the heart during routine cardiac electrophysiological studies. Congenital inferior vena cava anomalies are encountered rather infrequently during adult cardiac electrophysiology procedures. Their presence may preclude or pose technical challenges in the successful performance of catheter ablation procedures.^1^,^2^

## CASE REPORT

### Case-1:

A 30 year old obese gentleman presented with longstanding history of recurrent episodes of narrow QRS complex tachycardia which required frequent emergency room visits for medical treatment. A cardiac electrophysiology (EP) study was scheduled. Right femoral vein access was obtained. As a rule in our lab, after gaining access through the Seldinger technique, we check the position of the guide wires so that they are aligned to the right of the spine which confirms the venous route to the heart before placing sheaths. In our patient, the guide wire was noticed to track to the left of the spine; an inadvertent arterial access had to be excluded. Another femoral venous access was taken and 0.032 inch long J -tipped wire was advanced to track and assess the venous route. The long wire was noticed to cross to the left of the midline, then coursed up and roughly at the level of the renal veins curved back to the right of the spine before finally entering the right atrium through the inferior vena cava (IVC) route. Venography was performed through femoral venous sheath which highlighted the left-sided IVC route to the right atrium. (Fig.1-A).

**Fig.1-A:**
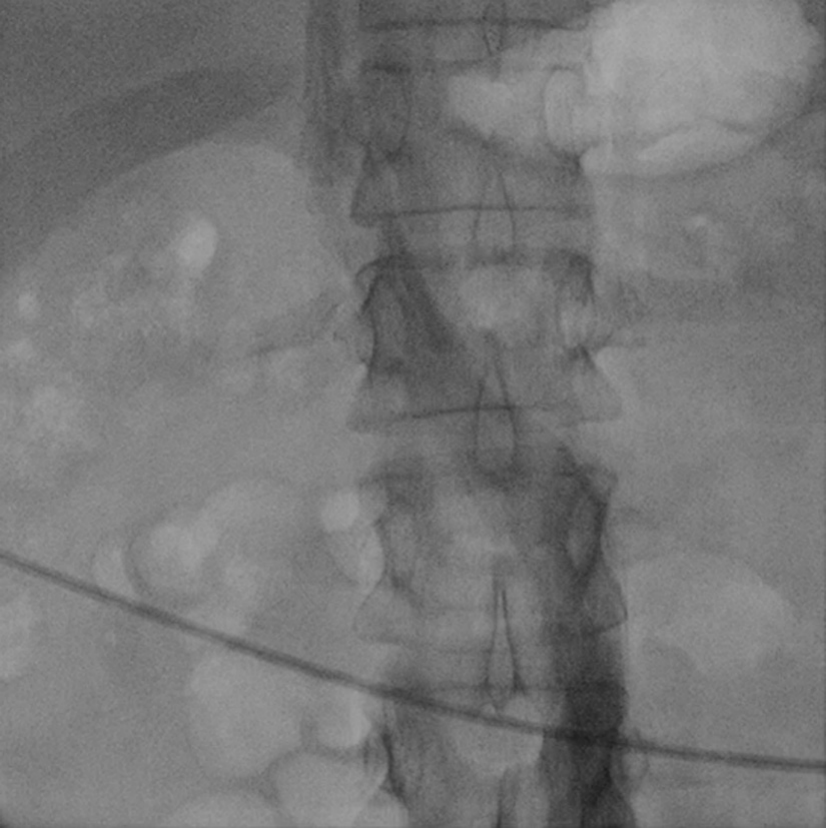
Venography via the right femoral vein demonstrating contrast flow into the left-sided inferior vena cava and tracking up into to the heart.

Next, we placed three femoral venous sheaths and advanced a steerable ablation catheter for positioning at His bundle region and two quadripolar non-steerable electrophysiology catheters to the right atrium and ventricle. We encountered some difficulty negotiating the tortuous IVC course at the level of the renal veins. (Fig.1-B). A decapolar catheter was advanced from the left subclavian vein for coronary sinus cannulation. During programmed electrical stimulation, typical slow-fast atrio-ventricular nodal re-entry tachycardia (AVNRT) was easily induced. Successful radio-frequency (RF) ablation of slow pathway region with a 7 F, 4 mm tip Medtronic Marinr MCXL RF ablation catheter at 40 Watts and 60^o^ C was performed. The tachycardia was rendered non-inducible after two RF lesions. The following day of procedure, abdominal contrast CT scan was performed and that revealed a left-sided IVC draining into the right atrium in this unusual case. (Fig. 2).

**Fig.1-B:**
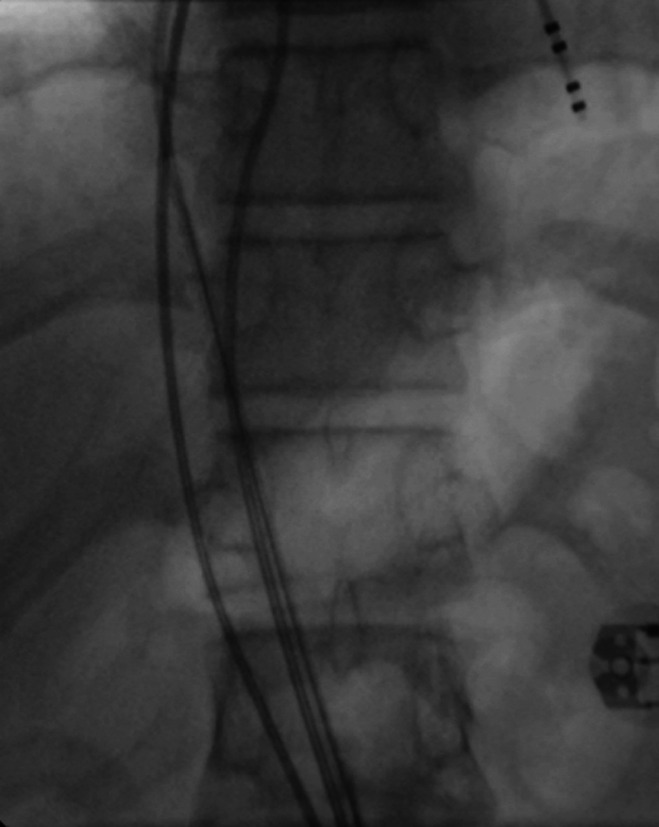
Fluoroscopy A.P. view showing the course of two non-steerable quadripolar electrophysiology catheters and a deflectable 7 F radiofrequency ablation catheter ascending up the right femoral vein enroute the left sided IVC to the heart.

**Fig.2:**
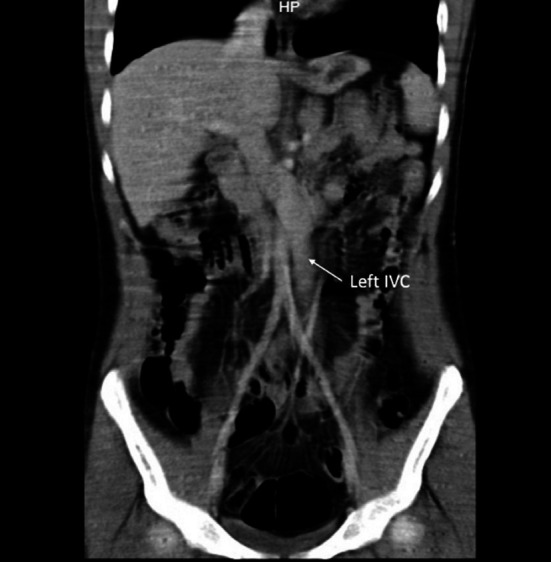
Contrast enhanced C.T. scan of abdominal vessels revealing the course of the left sided inferior vena cava (arrow).

### Case-2:

A 21 year old boy with Wolff-Parkinson-White syndrome and recurrent supraventricular tachycardia presented for cardiac electrophysiology study. Right femoral vein access was obtained. Similar to Case 1, we encountered a left-sided IVC course to the right atrium. With slight manipulation for tortuous venous anatomy, 7 F 4mm tip Medtronic Enhancr II RF ablation catheter and then non-steerable EP catheters were advanced to the right atrium and ventricle. Left subclavian venous access was used for coronary sinus cannulation. During programmed electrical stimulation, an orthodromic atrioventricular reciprocating tachycardia involving a right posterior accessory pathway was induced, that was mapped at 6 0’clock on the tricuspid annulus and successfully ablated during sinus rhythm using radiofrequency energy. Contrast CT scan of abdomen done the other day confirmed left-sided IVC.

## DISCUSSION

Development of the IVC is the result of a dynamic process involving the formation, regression and anastomoses of three parallel pairs of embryonic veins, i.e. post-cardinal, sub-cardinal and supra-cardinal veins. The final product of the remaining parts of these sets of veins is the adult IVC and iliac bifurcation.^3^ It is foreseeable that with this complex ontogeny of the IVC, anomalies of the IVC can occur. In order of incidence, the clinically important ones described are: duplication or double IVC (0.2-0.3%), transposition or left-sided IVC (0.2-0.5%) and interruption of IVC with azygous continuation (0.6%).^4^,^5^

Transposition or left-sided IVC results from the regression of the right supra-cardinal vein with persistence of the left supra-cardinal vein, which is one of the last veins to disappear. In majority of the cases, the left IVC crosses over to the right side via the left renal vein. The crossover is usually anterior but rarely posterior to the aorta.^6^ It then unites with the right renal vein to enter the right atrium in the usual fashion. Entire transposition of the IVC to the left with hemi-azygous continuation is extremely rare.^7^

The majority of IVC anomalies are asymptomatic, found incidentally during pre-operative imaging or during right heart catheterization. These anomalies may pose technical challenges for advancing temporary pacing wires, Swan Ganz catheters, performing right heart catheterization or placement of IVC filters.^8^ The left-sided IVC may also be associated with anomalies of the renal veins, therefore surgery performed in the retroperitoneum may entail risk to vascular structures if IVC anomalies are not suspected.^9^

EP studies and radiofrequency catheter ablation procedures are conventionally performed via the femoral venous approach as this approach is safe, highly effective and results in reduced radiation exposure to the patient and operator. Congenital anomalies of the IVC may limit accessing the right atrium via the femoral vein.^1^,^10^ In our cases, we encountered some maneuverability issue during advancing catheters, especially at the sharp angulation point where the left-sided IVC crossed over to the right at the level of renal veins, but that was successfully negotiated. A deflectable long sheath e.g. Agilis sheath, Abbott Inc. could have been used for catheter stability and support, but we feel that using our strategy of first maneuvering the deflectable ablation catheter, this resulted in relative straightening of the vessel which facilitated advancement of other catheters. Alternate venous access sites like the subclavian or internal jugular veins have been utilized to perform catheter ablation procedures in the presence of more complex IVC anomalies, precluding access to the right heart via the IVC.^2^ Herein we have described the first report of successful radiofrequency catheter ablation of slow pathway and right sided accessory pathway via the femoral vein approach in patients with a left-sided IVC.

## CONCLUSION

Congenital anomalies of inferior vena cava are rare occurrences. Suspicion and confirmation of venous anomaly is an important consideration to be recognized by an electrophysiologist while performing catheter ablation procedures. Catheter ablation of routine supra-ventricular tachycardias can be performed safely and successfully via femoral venous route in patients with IVC anomaly like the left-sided IVC; however, it is prudent to be aware of the technical difficulties and associated complications.

### Authors Contribution:

**RAA, MFK & ZM:** Collected patient data and were involved in manuscript writing.

**FQ:** did the manuscript review and final approval of version to be published.
